# Development of a novel molecular probe for the detection of liver mitochondrial redox metabolism

**DOI:** 10.1038/s41598-020-73336-1

**Published:** 2020-10-05

**Authors:** Md. Zahangir Hosain, Fuminori Hyodo, Takeshi Mori, Koyo Takahashi, Yusuke Nagao, Hinako Eto, Masaharu Murata, Tomohiko Akahoshi, Masayuki Matsuo, Yoshiki Katayama

**Affiliations:** 1 Graduate School of Systems Life Sciences, Kyushu University, 744 Motooka, Nishi-ku, Fukuoka, 819-0395 Japan; 2 Department of Radiology, School of Medicine, Gifu University, 1-1 Yanagido, Gifu, 501-1194 Japan; 3 Innovation Center for Medical Redox Navigation, Kyushu University, 3-1-1 Maidashi, Higashi-ku, Fukuoka, 812-8582 Japan; 4 Department of Applied Chemistry, Faculty of Engineering, Kyushu University, 744 Motooka, Nishi-ku, Fukuoka, 819-0395 Japan; 5 Center for Future Chemistry, Kyushu University, 744 Motooka, Nishi-ku, Fukuoka, 819-0395 Japan; 6 Center for Advanced Medical Innovation, Kyushu University, 3-1-1 Maidashi, Higashi-ku, Fukuoka, 812-8582 Japan; 7 International Research Center for Molecular Systems, Kyushu University, 744 Motooka, Nishi-ku, Fukuoka, 819-0395 Japan; 8 Center for Advanced Medical Innovation, Kyushu University, 744 Motooka, Nishi-Ku, Fukuoka, 819-0395 Japan; 9 Department of Biomedical Engineering, Chung Yuan Christian University, 200 Chung Pei Rd., Chung Li, 32023 Taiwan, ROC; 10 Department of Frontier Science for Imaging, School of Medicine, Gifu University, 1-1 Yanagido, Gifu, 501-1194 Japan

**Keywords:** Biological techniques, Imaging, Sensors and probes

## Abstract

Redox status influences the course of the inflammatory, metabolic, and proliferative liver diseases. Oxidative stress is thought to play a crucial and sustained role in the pathological progression of early steatosis to severe hepatitis, fibrosis, cirrhosis, and hepatocellular carcinoma. Oxidative stress induced by reactive oxygen species which are generated in the mitochondria can lead to chronic organelle damage in hepatocytes. Currently, the diagnosis of liver disease requires liver biopsy, which is invasive and associated with complications. The present report describes the development of a novel molecular probe, EDA-PROXYL, with higher reactivity and mitochondrial selectivity than standard carboxyl-PROXYL and carbamoyl-PROXYL probes. The membrane permeability of our probe improved in aqueous environments which led to increased accumulation in the liver and interaction of EDA-PROXYL with the carnitine transporter via the amine (NH_3_^+^) group further increased accumulation. This increased mitochondrial sensitivity and enhanced accumulation highlight the potential of EDA-PROXYL as a molecular probe for determining metabolic reactions of the mitochondria. Thus, this novel probe could be a tool for the evaluation of redox status of the mitochondria to assess the degree of liver injury and, ultimately, the response to pharmacological therapy.

## Introduction

Liver disease is the most predominant and significant cause of morbidity and mortality in the world and includes a range of conditions from early steatosis to severe hepatitis, fibrosis, cirrhosis, and hepatocellular carcinoma^1^. Liver disease can be influenced by risk factors such as obesity, alcohol, drugs, and exposure to other toxins^2^. As the liver is the main organ involved in detoxification and nutrient metabolism, it is more vulnerable to oxidative stress caused by the presence of toxic materials and metabolites in the body^3^. Therefore, oxidative stress plays an important role in the pathophysiology of various liver diseases.

Oxidative stress increases mitochondrial transcription and replication^4^. The electron transport chain is blocked in damaged mitochondria, resulting in the accumulation of reactive oxygen species (ROS)^5^ and increase in free fatty acid β-oxidation which promotes the accumulation of lipids. Increased ROS production initiates a self-sustaining loop that causes chronic organelle damage such as damage to mitochondrial DNA and iron-sulphur cluster enzymes^6,7^. Malondialdehyde and 4-hydroxynonenal are produced from lipid peroxidation and inhibit cytochrome c oxidase in mitochondrial complex IV of the hepatocytes^8^. In fact, various ultrastructural abnormalities and mitochondrial dysfunction are observed in hepatic disorders and some studies have revealed that redox status in the context of liver injury is related to the condition of the mitochondria as these are the main source of ROS^9–11^. Steatohepatitis is a type of liver disease that can progress to cirrhosis or end-stage liver disease, and may ultimately result in hepatocellular carcinoma^12^. Oxidative stress is thought to play a key role in the progression of steatohepatitis to fibrosis, cirrhosis, and liver cancer^1^. Currently, diagnosis of liver disease requires liver biopsy^13,14^ which has some drawbacks as it is an invasive procedure which may cause bleeding or infection and has been suggested to be prone to sampling errors and misdiagnosis^15,16^. Therefore, the development of a non-invasive technique is urgently required for specific diagnosis of hepatic disorders; the use of molecular probes is a promising approach.

In a previous report, we showed that early changes in mitochondrial redox metabolism can be detected using a redox-sensitive probe carbamoyl PROXYL (CmP) with in vivo dynamic nuclear polarization-magnetic resonance imaging (DNP-MRI)^17^. The MRI signals from nuclei are increased by the use of DNP^18^ as this increases the electron paramagnetic resonance (EPR) frequency of free radicals prior to applying the pulse sequence of MRI, thus increasing the image intensity in tissues where free radicals are present. Nitroxyl radicals modulate the redox reaction and the rate of reduction relies upon the redox status which is influenced by factors such as the level of production of ROS and antioxidant activity. Some studies have demonstrated the utility of nitroxyl radicals to obtain information on redox status^19–21^, tissue partial pressure of oxygen^22,23^, pH^24^, and proteolytic activity^25^ through in vivo DNP-MRI in living mice.

The carboxyl-PROXYL (CxP) and CmP that were used as DNP contrast agents in the present study are blood-stable radical compounds used for different biophysical and biomedical experiments^17,26,27^. Previous studies have shown that CxP is membrane impermeable while CmP is membrane permeable^28^, which might influence the redox reaction of these agents with ROS. In addition, CmP may be reduced by mitochondrial redox metabolism in the inner membrane^17,29^. Furthermore, cytoplasmic components such as antioxidants and constituents of the electron transfer system of the mitochondrial membrane eliminate radicals from these probes^30,31^. Although the mitochondrial redox status was assessed using CmP^17^, a stronger DNP signal would enable more precise and fast three-dimensional (3D) imaging for assessment of hepatic steatosis. To overcome the current problems in the field, we designed and synthesized a novel molecular probe, EDA-PROXYL, using the basic skeleton of CmP with the aim of creating a probe with high sensitivity to the mitochondria and enhanced accumulation in the liver. This novel probe could represent a therapeutic approach for the precise evaluation of the redox state of the mitochondria in the context of liver disease.

## Materials and methods

### Chemicals

We purchased 3-Carboxy-2,2,5,5-tetramethyl-1-pyrrolidine-1-oxyl (Carboxy-PROXYL) and 3-Carbamoyl-2,2,5,5-tetramethyl-1-pyrrolidine-1-oxyl (Carbamoyl-PROXYL) from Sigma-Aldrich (Saint Louis, MO, USA). Benzotriazol-1-yl-oxytripyrrolidinophosphonium hexafluorophosphate (PyBOP) was purchased from Merck. Potassium cyanide (KCN) and potassium hexacyanoferrate (III) (K_3_[Fe(CN)]_6_) were obtained from Wako Pure Chemical Industries (Osaka, Japan). All other chemicals used in this study were reagent-grade quality and commercially available.

### Synthesis of EDA-PROXYL

We dissolved CxP (1.00 g, 5.37 mmol) and PyBOP (2.79 g, 5.37 mmol) in dry tetrahydrofuran (50 mL) containing triethylamine (0.5 mL). The mixture was stirred for 30 min at room temperature. Ethylenediamine (2 mL, 74.9 mmol) was added and the mixture stirred for a further 24 h in a nitrogen atmosphere. The resulting mixture was purified using silica gel column chromatography (chloroform/methanol/triethylamine = 90/10/1). The compound of interest was identified by electrospray ionization mass spectrometry: m/z: calcd for C_11_H_23_N_3_O_2_ (M + H^+^), 229.2; found, 229.2. The compound was obtained as a pale-yellow liquid (yield: 37.7%).

### Animals and dietary treatments

Animal protocols and dietary treatments were as described in our previous report^17^. All animal experiments and protocols were approved by the Ethics Committee for Animal Experiments of Kyushu University and were conducted in accordance with the Guidelines of the Committee for Care and Use of Laboratory Animals, Kyushu University. Male C57BL/6 mice 5 weeks of age were purchased from Charles River Laboratories Japan, Inc. (Yokohama, Japan), and housed in temperature and light-controlled chambers (24 °C, 12 h/12 h light/dark cycle). Before starting experiments, all animals were acclimatised for 1 week and allowed water and appropriate food (MF diet, Oriental Yeast Co., Tokyo, Japan) ad libitum. At the age of 6 weeks, they were divided into five groups (n = 5), three groups received a normal diet and two were fed a methionine-choline-deficient (MCD) diet (Oriental Yeast Co., Tokyo, Japan) for 5 weeks to create a non-alcoholic steatohepatitis (NASH) model^17^. Mice were fasted overnight prior to any measurements.

### Evaluation of redox reaction of the probe with antioxidants

We used L-ascorbic acid to evaluate the redox reaction of the probe with antioxidants. We prepared 2.0 mM of each probe (CmP, CxP, or EDA-PROXYL) in phosphate-buffered saline (PBS). Next, a 20 mM solution of L-ascorbic acid sodium salt was prepared in PBS and both solutions were mixed at a ratio of 1:1, so that the final concentration of probe 1 mM and ascorbic was 10 mM. The time of mixing was taken as 0 min, and the electron spin resonance (ESR) spectrum was recorded every 1.5 min from 1.5 min to 15 min. Measurement parameters of X-band EPR were as follows: microwave frequency, 1 GHz; microwave power, 1 mW; the centre of the field, 336.2 mT; modulation width, 0.3 × 0.1 mT; sweep time, 60 s; sweep width, 5.0 mT; and time constant, 0.03 s.

### Assessment of probe metabolism in liver tissue using X-band electron paramagnetic resonance

For EPR, we used a similar method as described in our previous study^17^. In brief, liver tissue was excised from C57BL/6 N mice and the surface of the tissue washed with homogenate buffer containing 70 mM sucrose, 220 mM mannitol, 2 mM HEPES, 1 mM EGTA, 0.2% bovine serum albumin, 10 mM MgCl_2_ and 20 mM KH_2_PO_4_, pH 7.2. Then, homogenate buffer was added to liver samples at a ratio of 4:1 (w/w) buffer: liver followed by homogenization at 1,000 rpm. The resulting homogenate was centrifuged at 8500×*g* and 4 °C for 10 min to remove cell membranes. The supernatant was collected and centrifuged again at 10,000×*g* and 4 °C for 7 min to remove the mitochondria. The supernatant was recovered and used as the cytosol fraction. The reduction rate of probe radicals was measured by X-band EPR at 5 min after mixing of probes (50 μM) and liver homogenates. Furthermore, after the addition of KCN into homogenate solution and cytosol fraction, the radical concentrations were detected by X-band EPR. Measurement parameters for X-band EPR were as follows: microwave frequency, 1 GHz; microwave power, 1 mW; centre of the field, 336.3 mT; modulation width, 0.3 × 0.1 mT; sweep time, 60 s; sweep width, 5.0 mT; and time constant, 0.03 s.

### Quantitative analysis of oxidized and total probe levels in liver tissue

We dissolved CmP, CxP, and EDA-PROXYL separately in half saline to a final probe concentration of 150 mM. Overnight fasted C57BL/6 N mice received CmP, CxP, or EDA-PROXYL intravenously at a dose of 5 μL/g of body weight. After 5 min, the mice were sacrificed and the livers excised. Twice the liver volumes of half saline were added to each liver and livers were homogenized at 2,000 rpm. The concentration of radicals in the samples was measured using X-band EPR and the total concentration of CmP, CxP, or EDA-PROXYL was measured after the addition of ferricyanide. Measurement parameters of X-band EPR were as follows: microwave frequency, 1 GHz; microwave power, 1 mW; the centre of the field, 336.3 mT; modulation width, 0.3 × 0.1 mT; sweep time, 60 s; sweep width, 5.0 mT; and time constant, 0.03 s.

### Redox metabolic imaging using in-vivo dynamic nuclear polarization-magnetic resonance imaging

Redox metabolic imaging was carried out using a low magnetic field for in vivo DNP-MRI as described in our previous report^17^. In brief, a rectangular, one-turned, curved-surface coil (longitudinal length, 20 mm; lateral length, 32 mm) was used for EPR illumination. During the procedure, the body temperature of the mice was kept at 37 ± 1 °C with a heating pad. Mice were anesthetized with 2% isoflurane and then fixed in a dorsal recumbent position on a customized holder using adhesive skin tape. The holder was placed in centre of the resonator and in vivo DNP-MRI scanning of the upper abdomen was performed immediately after intravenous injection of CmP, CxP, or EDA-PROXYL. Pharmacokinetic DNP-MRI images of mice were taken at 1-min intervals until 10 min after administration. Normal MRI images with 1 and 10 accumulations were taken without EPR irradiation. The in vivo redox map was obtained from the slope of the enhanced DNP image intensity of each pixel from four pharmacokinetic images using a custom Excel macro program. Scanning conditions for in vivo DNP-MRI were as follows: power of EPR irradiation, 7 W; flip angle, 90°; repetition time (T_R_) × echo time (T_E_) × EPR irradiation time (TEPR), 500 × 25 × 250 ms; number of accumulations, 2; slice thickness, 100 mm including the whole thickness of the mouse; phase-encoding steps, 32; field of view (FOV), 40 × 40 mm; and matrix size, 64 × 64 after reconstruction. The in vivo DNP-MRI data of both normal and NASH-model mice were analysed using Image J software.

## Results and discussion

**Figure 1:**
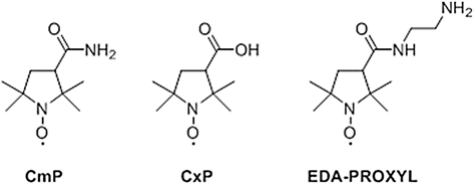
Chemical structures of the proxyl derivatives used in this study.

The radical compounds, CmP and CxP are reported to show as contrast agent for DNP-MRI or EPR in diagnosis of many diseases, based on its functionality tissue redox activity^28^. Although, these compounds have contrast activity, but the cell membrane permeability and tissue redox activity of CxP are lower compared to CmP^27^. CmP has moderate reactivity and very low toxicity in living tissue^32^. Therefore, to obtain 3D fast imaging of redox metabolism for the diagnosis of liver diseases including steatohepatitis, considerable accumulation of nitroxyl probe in the liver is required. We aimed to synthesize a novel probe using the basic skeleton of CmP to increase the rate of liver accumulation and thus enhance the radical signal. After intravenous administration, nitroxyl probes are distributed throughout different organs, but rapid uptake of the probe with liver parenchymal cells is required to achieve sufficient distribution in liver tissue for imaging. Therefore, two properties are considered for the selection of our probe. The increased selectivity for hepatic cells and enhanced accumulation property in liver tissue, targeting the transporters on the cell membrane. Various transporters exist in hepatocyte cell membranes which efficiently translocate functional molecules from the blood into hepatocytes^33^. We developed the EDA-PROXYL probe (Fig. 1) with the expectation that uptake would occur via the organic cation transporter-1 (OCT 1) system which plays an important role in transporting bioactive molecules across the inner membrane of mitochondria^34^. In addition, we investigated the sensitivity of our novel probe for liver tissue with the goal of enabling detection of the redox state of the mitochondria.

**Figure 2:**
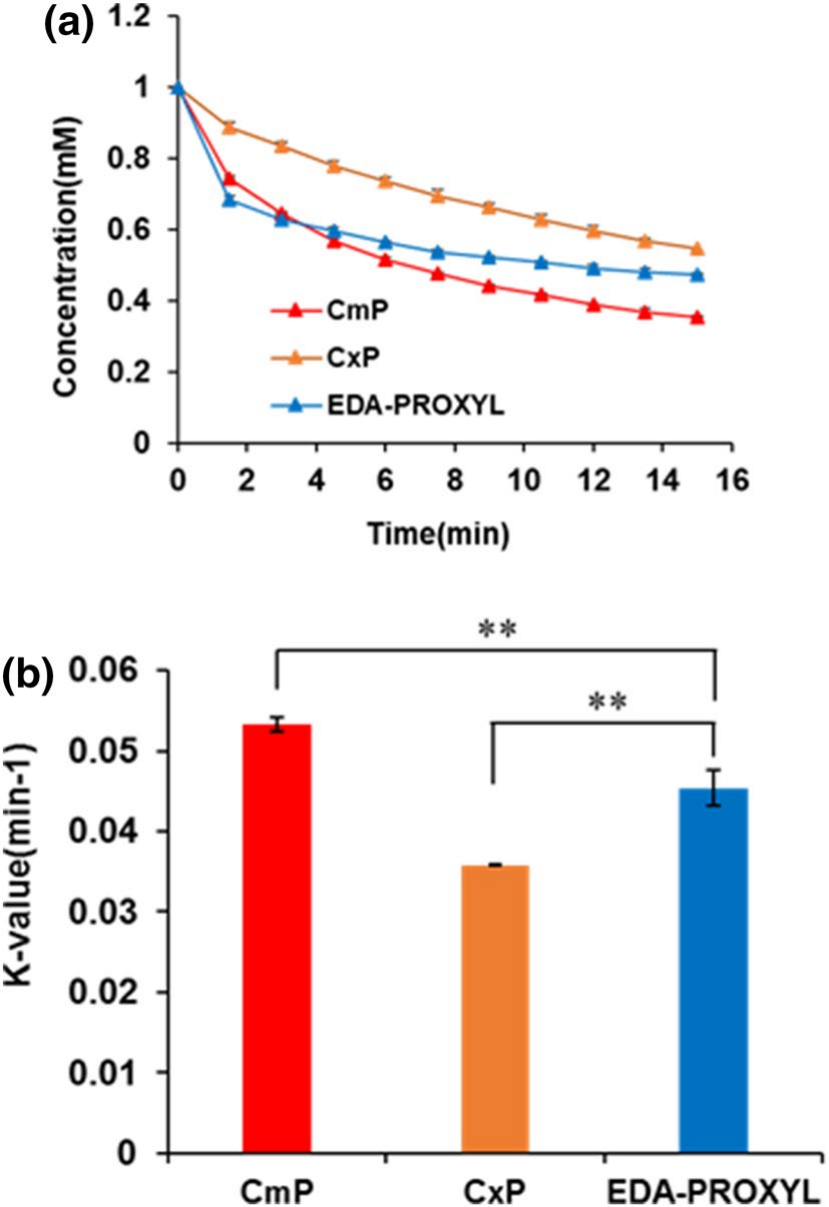
(**a**) Temporal changes in the radical concentrations of carbamoyl PROXYL, carboxyl PROXYL, and EDA-PROXYL due to reduction by ascorbic acid over the time course of 1 to 15 min. Data are presented as mean ± standard deviation (n = 3 per group). (**b**) Reduction rates of the three probes through reaction with ascorbic acid over the time course of 1 to 15 min. Data are presented as mean ± standard deviation (n = 3 per group). *p < 0.05; **p < 0.01.

Ascorbic acid acts as a reducing agent in various enzymatic and non-enzymatic reactions^35^ as well as playing a significant role in the reduction of nitroxyl radical in hepatocytes^36^. Many nitroxyl probes react with ascorbic acid losing their free radical through reduction. Our evaluation of the redox reduction of nitroxyl radicals of CmP, CxP and EDA-PROXYL with ascorbic acid using X-band EPR revealed the redox reaction between CxP and ascorbic acid to be slower than either CmP and EDA-PROXYL with ascorbic acid. In contrast, the redox reactions of CmP and EDA-PROXYL were similar between 1 to 15 min, but the rate of reduction of EDA-PROXYL was slower than that of CmP (Fig. 2a, b).

**Figure 3:**
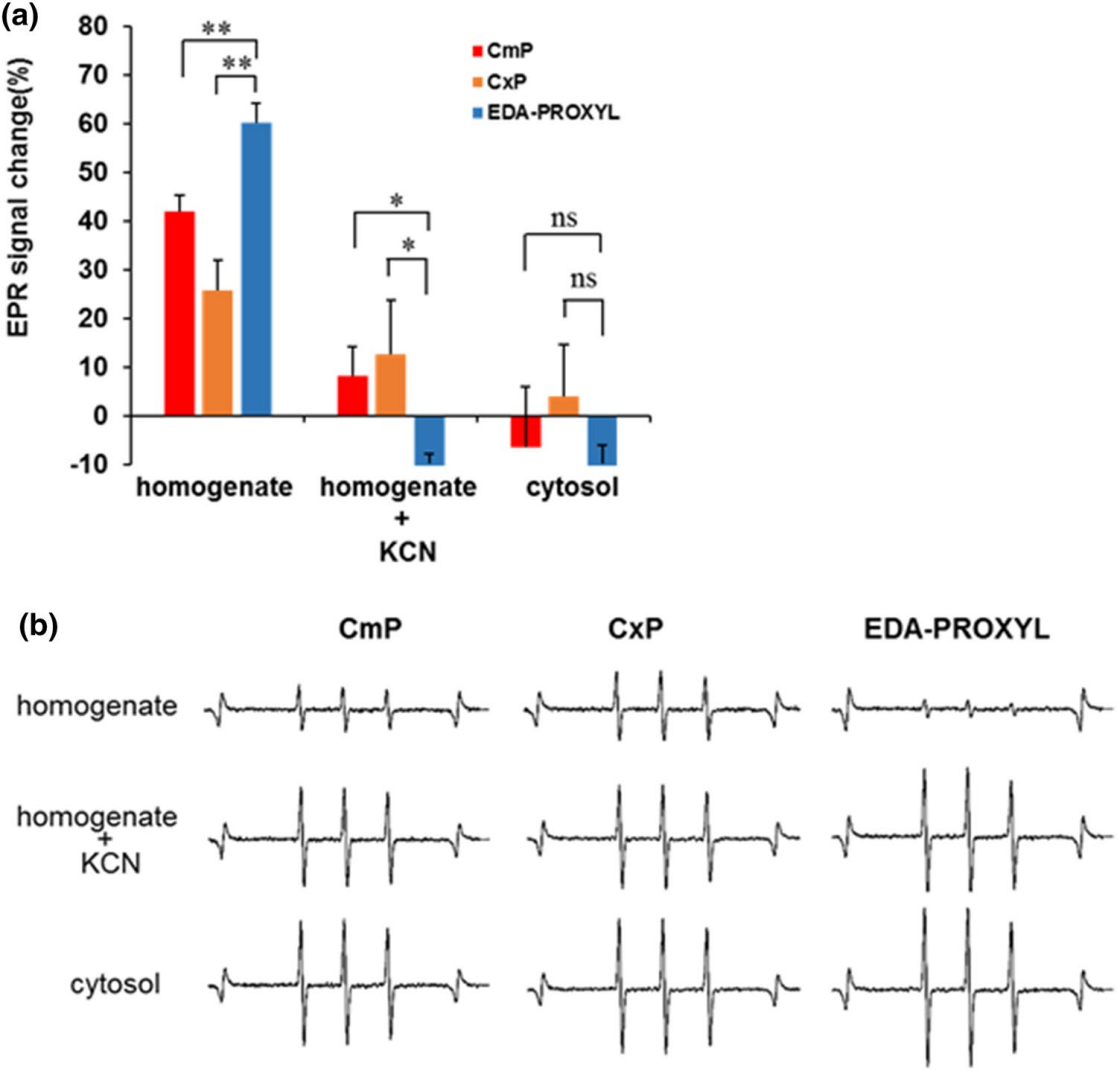
(**a**) Graph illustrating the rates of reduction of carbamoyl PROXYL, carboxyl PROXYL, and EDA-PROXYL in liver tissue homogenate as measured by X-band electron spin resonance. The relationship between mitochondrial status and reactions of the probe were confirmed. The reduction rates were measured again after the addition of 200 mM potassium cyanide. Reduction rates of each probe after incubation with the cytosol fraction were also measured in the same way. Data are presented as mean ± standard deviation (n = 5 per group). *p < 0.05; **p < 0.01; ns, not significant. (**b**) Typical 5-min electron spin resonance signal attenuations of the three probes in fivefold diluted liver tissue homogenate solution.

We evaluated redox metabolism by analysing the redox reaction between the probes and liver homogenates using X-band EPR. The EPR signal of all the probes was reduced in the freshly prepared liver homogenates at 5 min, and the EPR signals of CmP and CxP were significantly lower than that of EDA-PROXYL (Fig. 3a, b). The addition of KCN, an inhibitor of complex IV in the mitochondrial electron transfer chain (ETC)^37^, suppressed radical metabolism in all groups of normal mice but more in mice who received EDA-PROXYL, may result from combined effect of KCN and basicity of amine (NH_3_^+^) group present in EDA-PROXYL. Furthermore, CmP, CxP, and EDA-PROXYL were not reduced by the cytosol solution (which did not contain mitochondria) (Fig. 3a, b), indicating that the redox reaction of these probes does not occur in the cytosol. These results confirm that mitochondrial redox metabolism, especially the electron transport chain in the mitochondria, is crucial for the reduction of all the probes tested. Previous reports have shown the pathology of liver disorders to be related to mitochondrial abnormalities^38,39^. Direct monitoring of EDA-PROXYL in the mitochondria might be a reliable method for evaluating mitochondrial function and the maintenance of this function in redox status.

**Figure 4:**
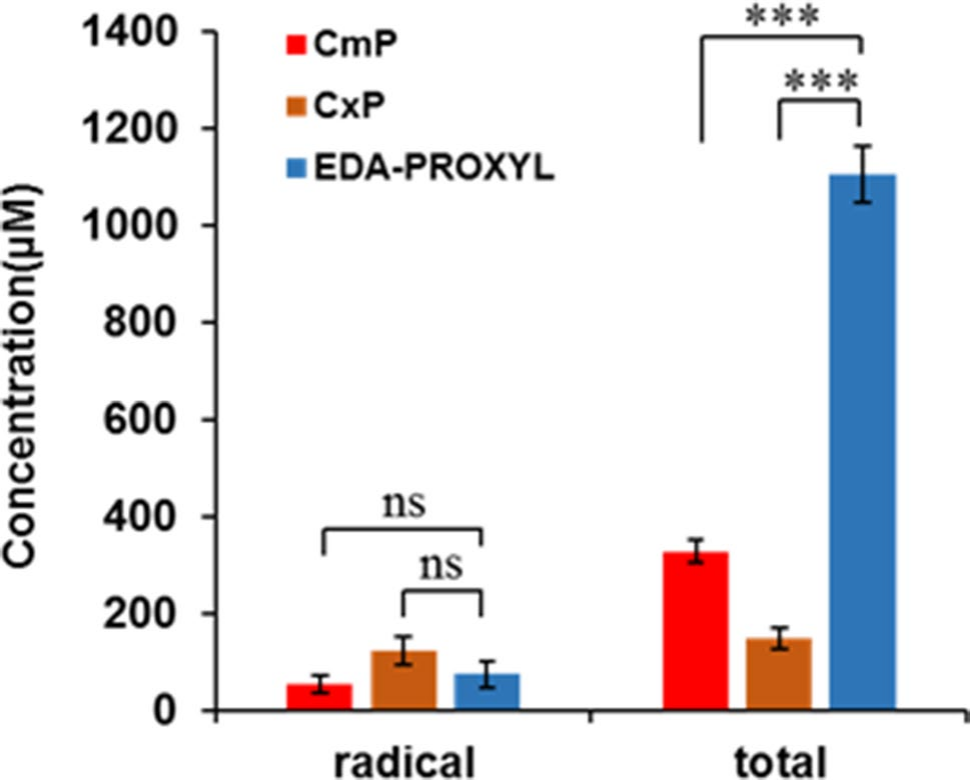
Analysis of the oxidised forms of carbamoyl PROXYL, carboxyl PROXYL, and EDA-PROXYL and total (oxidized plus reduced form) of each probe in the liver at 15 min after administration. The electron paramagnetic resonance signal intensity of probes in the liver homogenates were measured using X-band electron spin resonance (n = 5). Total amounts of each probe were measured after re-oxidation with potassium ferricyanide. Data are presented as mean ± standard deviation (n = 5 per group). *p < 0.05; ***p < 0.001; ns, not significant.

The accumulation of EDA-PROXYL in the liver after intravenous administration is critical for the diagnosis of liver diseases, as sufficient DNP signal is required for adequate imaging. We assessed the extent of liver accumulation of our novel probe compared with that of CmP and CxP. Figure 4 shows the liver accumulation of the probe after intravenous administration in mice. The total concentration of each nitroxyl probe (reduced + oxidized) was calculated by analysing the oxidation of reduced probe by adding potassium ferricyanide. This showed that the accumulation of CxP^40^ in the liver was significantly reduced compared with the membrane-permeable probe CmP^41^ and EDA-PROXYL showed significantly higher accumulation in the liver compared with both CmP and CxP (by about 3- and fivefold, respectively). This suggests that the transport of EDA-PROXYL from blood into liver cells is more efficient than that of CmP and CxP. We also found that the accumulation of EDA-PROXYL in NASH-model mice was significantly different to that of CmP (Supplementary Fig. S1). Therefore, increased liver accumulation of EDA-PROXYL is universal, possibly via the cation transporter system in the mitochondrial membranes of hepatocytes.

**Figure 5:**
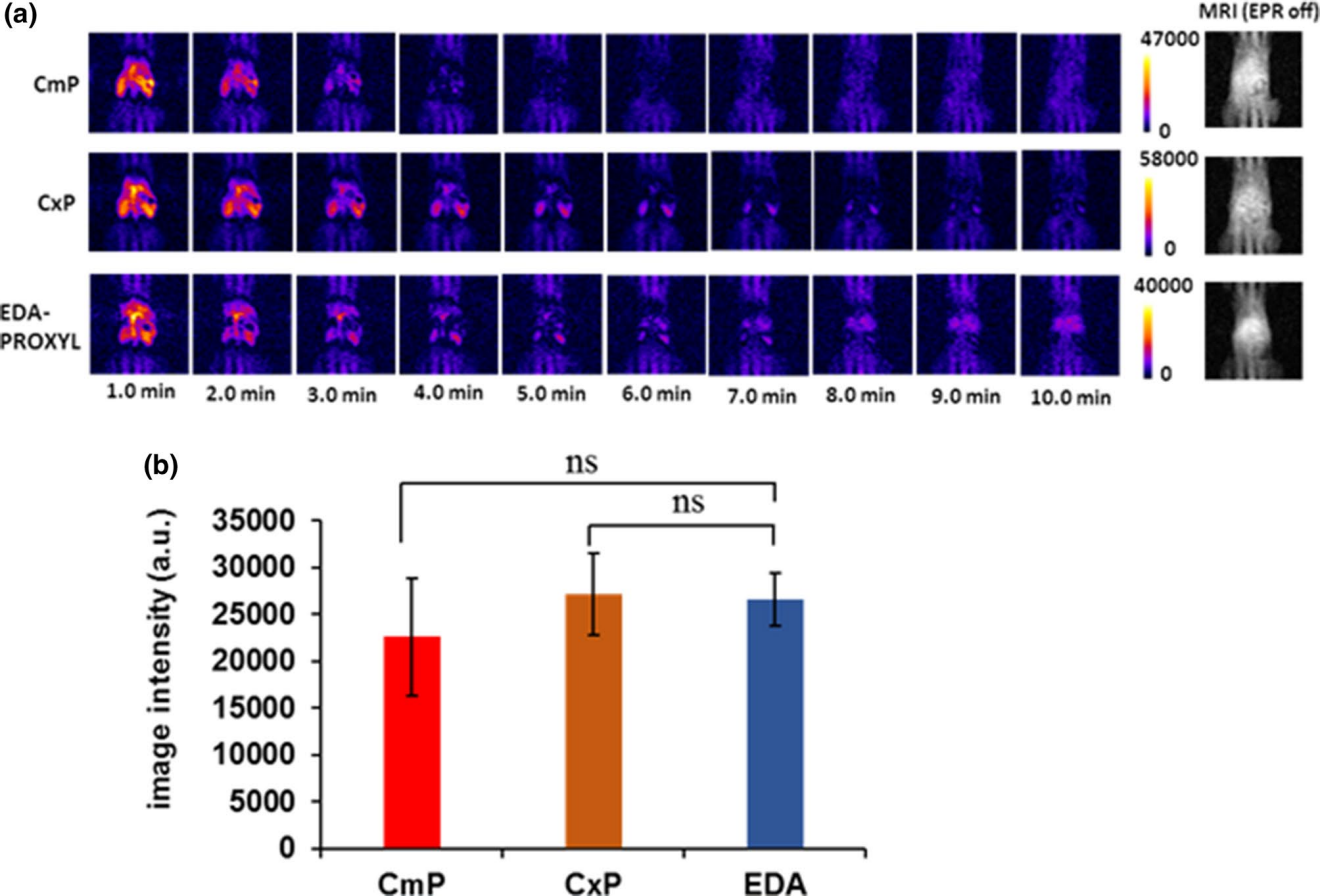
(**a**) In vivo kinetic dynamic nuclear polarization-magnetic resonance images of the upper abdomen regions of mice after intravenous injection of carbamoyl PROXYL, carboxyl PROXYL, or EDA-PROXYL. The in vivo DNP-MRI data were analysed using Image J software^51^ (n = 5 per group), (**b**) Image intensity 1 min after intravenous injection of each probe. Data are presented as mean ± standard deviation (n = 5 per group).

Our in vivo DNP MRI study of the upper abdomen of living mice after intravenous administration of the probes revealed clear DNP enhancement in this region immediately after intravenous administration for all probes. Furthermore, the distributions and metabolic rates in the liver were different for each probe (Fig. 5a, b). The DNP enhancement of EDA-PROXYL showed the distribution throughout the liver region; the decay of image intensity was faster than that of the other two probes although the initial image intensity in the liver region was not different between the probes. The distribution and metabolic rates of CmP and EDA-PROXYL were also checked in NASH model and the result showed a higher distributions and metabolic rates for EDA-PROXYL in the liver tissue compared to CmP (Supplementary Fig. S2). Various types of transporters such as organic anion transporter (OATP), OCT 1, and OCTN 1^42,43^ exist on the basolateral membrane of hepatocytes of both mice and humans. The carnitine/organic cation transporter OCTN1, classified as a xenobiotic transporter, is ubiquitously expressed in the body^44,45^ and transports various therapeutic agents including organic cations and zwitterions^44,46,47^. This transporter is functionally expressed in mouse liver tissue^48–50^ and we speculated that EDA-PROXYL was mainly distributed in the liver region due to the presence of the carnitine-based transporter system. In contrast, CxP did not accumulate significantly in the liver due to its lower membrane permeability (Fig. 5a, b)^28^ and rapid reduction within the circulating blood caused by the interaction with several redox factors released from tissues^27^.

## Conclusions

We have developed and evaluated a novel molecular probe, EDA-PROXYL, which can be used to detect mitochondrial redox status in the context of liver disease. The membrane permeability of EDA-PROXYL improved in aqueous environments, reflected by the increased accumulation in liver tissue compared with CmP. The increased sensitivity and enhanced accumulation of EDA-PROXYL highlights its potentiality for determination of the redox status of mitochondria. Thus, this novel probe could be a useful and potential therapeutic approach for precise detection of redox metabolism in the mitochondria for the diagnosis of liver disease.

## Supplementary information

Supplementary Information.
