# Supplementary material for: Development of a novel molecular probe for the detection of liver mitochondrial redox metabolism
Source: Sci Rep. 2020 Oct 5;10:16489. doi: 10.1038/s41598-020-73336-1 (PMC7536409; doi:10.1038/s41598-020-73336-1)
Supplement: Supplementary file 1 — Supplementary Information. [file 41598_2020_73336_MOESM1_ESM.docx]

**Supplementary Information**

**Development of a novel molecular probe for the detection of liver mitochondrial redox metabolism**

Md. Zahangir Hosain, Fuminori Hyodo, Takeshi Mori, Koyo Takahashi, Yusuke Nagao, Hinako Eto, Masaharu Murata, Tomohiko Akahoshi, Masayuki Matsuo and Yoshiki Katayama

^
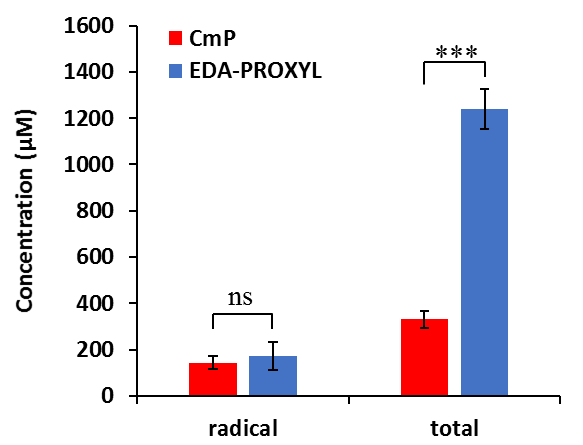
^

**Supplementary Figure S1**. Analysis of the oxidised form of CmP and EDA-PROXYL and total (oxidised and reduced form) of CmP and EDA-PROXYL in NASH model mice liver. The probe intensity was measured by using X-band electron spin resonance with homogenates of liver tissue after intravenous injection in mice (n = 5). Total CmP and EDA-PROXYL were measured after re-oxidative treatment with Potassium ferricyanide. Results represent the mean ± SD (n = 5 per group). ***p < 0.001; ns, not significant


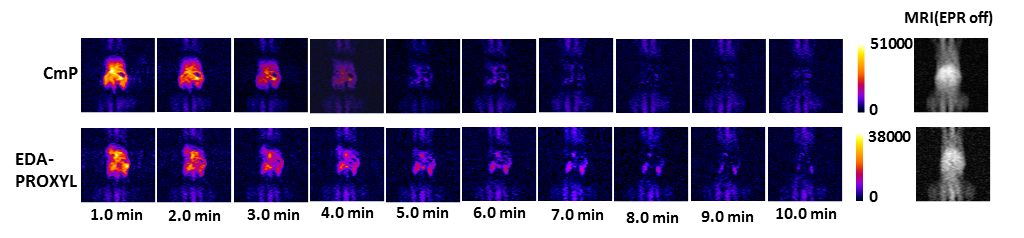


**Supplementary Figure S2.** *In vivo* kinetic dynamic nuclear polarization-magnetic resonance images of the upper abdomen regions of mice after intravenous injection of carbamoyl PROXYL and EDA-PROXYL in NASH model mice (n = 5 per group)
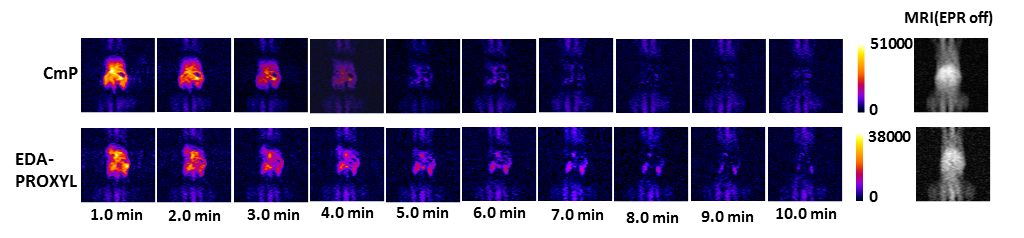


**Supplementary Figure 2.** Temporal changes in DNP-MRI after intravenous injection of CmP and EDA-PROXYL in MCD mice (n = 5, each group)
